# Efficacy of Fufang E'jiao Jiang in the Treatment of Patients with Qi and Blood Deficiency Syndrome: A Real-World Prospective Multicenter Study with a Patient Registry

**DOI:** 10.1155/2023/3179489

**Published:** 2023-02-03

**Authors:** Mengjie Zhao, Mengli Xiao, Jiake Ying, Panbo Qiu, Hongxian Wu, Qin Tan, Shiliang Chen, Lulu Zhang, Sanfei Shi, Guida Li, Yang Zhao, Fang Lu

**Affiliations:** ^1^Graduate School of Beijing University of Chinese Medicine, Beijing 100029, China; ^2^NMPA Key Laboratory for Clinical Research and Evaluation of Traditional Chinese Medicine, Xiyuan Hospital, China Academy of Chinese Medicine Sciences, Beijing 100091, China; ^3^National Clinical Research Center for Chinese Medicine Cardiology, Xiyuan Hospital, China Academy of Chinese Medicine Sciences, Beijing 100091, China; ^4^Zhongshan Hospital, Fudan University, Shanghai 200032, China; ^5^Shucheng Hospital of Traditional Chinese Medicine, Shucheng, Anhui 231300, China; ^6^Liaocheng People's Hospital, Liaocheng, Shandong 252000, China; ^7^Pan'an County TCM Hospital, Panan, Zhejiang 322300, China; ^8^Feixian People's Hospital, Feixian, Shandong 273400, China

## Abstract

**Objective:**

This nationwide, multicenter prospective observational study with a patient registry was designed to evaluate the efficacy of Fufang E'jiao Jiang (FEJ) in Chinese patients with Qi and blood deficiency syndrome (QBDS).

**Methods:**

QBDS patients were consecutively recruited from 81 investigational sites in China from July, 2019, to December, 2020. Patients who met the eligibility criteria were enrolled in a prospective registry database. Baseline characteristics and changes in scores on the traditional Chinese medicine (TCM) symptom evaluation scale for Qi and blood deficiency, the clinical global impression (CGI) scale, the fatigue scale-14 (FS-14), and the Pittsburgh sleep quality index (PSQI) were analyzed to determine the clinical efficacy of FEJ.

**Results:**

A total of 3,203 patients were recruited. The average remission rate (i.e., the sum of the cure rate and improvement rate) of the 20 symptoms of QBDS was 92.49% after 4 weeks of FEJ treatment, which was higher than at baseline; the rate increased to 94.69% at 8 weeks. The CGI scale revealed that the number of total remissions at 4 and 8 weeks was 3,120 (97.41%) and 415 (100%), respectively. The total FS-14 scores decreased by 1.67 ± 4.11 (*p* < 0.001) at 4 weeks and 1.72 ± 3.09 (*p* < 0.001) at 8 weeks of treatment. The PSQI scores were 6.6 ± 4.7 and 6.52 ± 3.07 at 4 and 8 weeks, respectively, which were significantly lower than the baseline scores (*p* < 0.001; *p* = 0.0033). Both the subhealth fatigue (SF) and iron deficiency anemia (IDA) groups showed significantly improved clinical symptoms of QBDS (*p* < 0.01). Between-group comparisons revealed significantly greater improvements in FS-14 and PSQI scores in the SF group than in the IDA group (*p* < 0.05). A multivariate logistic regression analysis showed that disease course, FS-14 score at baseline, and four-week FEJ doses were independent risk factors for the degree of symptom relief in QBDS patients (*p* < 0.05).

**Conclusion:**

In real-world settings, FEJ has a promising effect in treating QBDS and can significantly improve the severity of its symptoms.

## 1. Introduction

Qi and blood deficiency is a concept in the theory of traditional Chinese medicine (TCM) that refers to the deficiency of Qi in the organism or the diminished function of Qi combined with a blood deficiency that makes the organs and tissues lose the nourishment of Qi and blood, resulting in the corresponding weakening disease [1]. The main clinical manifestations of Qi and blood deficiency are dizziness, weakness, spontaneous sweating, shortage of Qi, slurred speech, pallor, palpitations, and insomnia [2]. Qi and blood deficiency syndrome (QBDS) is mainly seen in patients with chronic diseases such as anemia, leukopenia, and malignant tumors. Epidemiological studies show that approximately 75% of the global population is considered to be subhealthy, and healthy and ill people account for 5%, and 20% of the remaining individuals, respectively [3]. In China, healthy and ill people each account for 15% of the population, and subhealthy people account for 70%, which encompasses more than 900 million individuals [4]. Although subhealth does not cause organic pathology in the short term, it does seriously affect quality of life. Without proper intervention, it may increase the risk of diabetes, cardiovascular and cerebrovascular diseases, cancer, and other diseases [5].

There are no effective treatment modalities for subhealth states [6]. For anemia and leukopenia caused by radiotherapy for malignant tumors, treatment is usually carried out by iron supplementation, blood transfusion, hematopoietic stem cell migration, and immunosuppression subsequent to certain treatments, but the treatment efficacy is unstable, and those conditions easily relapse. Moreover, there are many side effects from these treatments [7–11].

Fufang E'jiao Jiang (FEJ) is a patented Chinese medicine that has been marketed in China to replenish Qi and nourish blood, and it has been approved by the China Food and Drug Administration (CFDA). FEJ is a Chinese herbal compound preparation composed of Ejiao (Asini Corii Colla), red ginseng, Radix Rehmanniae, Radix Codonopsis pilosula, and Hawthorn, of which Asini Corii Colla is the primary active ingredient [12]. It is mainly used to treat dizziness, palpitations, insomnia, poor appetite, leukopenia, and anemia caused by deficiency of Qi and blood [13–18]. However, there are few real-world studies on using FEJ for treating QBDS, and there are also no real-world studies on using FEJ in China or Asia. Therefore, we targeted our survey to the FEJ market with the aim of further exploring the potential clinical value of the prescription by observing the actual application and efficacy of FEJ marketed in a real-world setting.

## 2. Methods

### 2.1. Study Design

This is a multicenter, prospective, single-arm, observational real-world case study with a patient registry. From July, 2019, to December, 2020, this study recruited patients with QBDS from 81 study sites (including 19 hospitals, 35 pharmacies, 17 outpatient clinics, and 10 community medical service centers) in 6 cities in China (including Anhui, Beijing, Jiangsu, Henan, Shandong, and Shanghai). The study was conducted in accordance with the ethical principles of the Declaration of Helsinki and in accordance with good clinical practice guidelines and applicable local regulatory requirements. The trial protocol was approved by the Ethics Committee of Xiyuan Hospital, China Academy of Chinese Medicine Sciences (2019XLA027-2), which was accepted by the primary study site and all other centers. It was also registered with the Chinese Clinical Trials Registry (ChiCTR1900025328). This study is reported following the guidelines for the Strengthening the Reporting of Observational Studies in Epidemiology (STROBE) [19] (Supplementary Table 1).

FEJ, as used in the study, was the product of Shandong Dong-E-E-Jiao Co., Ltd. (Shandong, China) with batch No. Z20083345. The ingredients used in the preparation of FEJ include Asini Corii Colla, red ginseng, Radix Rehmanniae, Radix Codonopsis pilosula, and hawthorn. The standard dose of FEJ was 20 ml. The reference dosing regimen is one tablet per dose, administered orally three times daily. Adjusting the dosing regimen (reduction or increase in frequency and quantity) and discontinuing the drug is at the patient's discretion.

### 2.2. Participants

Patients who met the inclusion and exclusion criteria were consecutively recruited at the registration site. The inclusion criteria were as follows: meeting the TCM diagnostic criteria for QBDS [20] (Supplementary Table 2), taking FEJ, and agreeing to participate in the study. The exclusion criteria were as follows: contraindications to FEJ, such as allergy to FEJ or any excipients; participation in other clinical trials; and refusal to follow-up. We conveyed the details of this study to all participants at each study center. Patients who met the eligibility criteria were prospectively registered on the network (Electronic Data Capture, EDC). All enrolled patients or their authorized guardians signed an informed consent form before participation. Some selection bias may have accompanied patient selection of involvement, but we did not arbitrarily select patients. All patient details were deidentified simultaneously.

### 2.3. Data Collection

All data, including age, sex, clinical characteristics, symptom remission, concomitant diseases, and concomitant medications, were collected using an online mobile platform. All centers collected data according to our institution's uniform prespecified protocols. In addition, an EDC system was constructed for this project. Data are collected at each center, recorded on a case report form (CRF), and entered into the clinical trial EDC system by specially trained staff.

Patients in this study received FEJ according to local practice and the best interests of individuals. Consistent with the true observational nature of the study, patients included in the study were not involved in any treatment decisions because they were treated without any additional protocols. The treatment choice had to be made before the patient was enrolled in the study. Patients were enrolled and completed follow-up information as needed, which the researcher collected after 4 weeks of treatment. After completing the 4-week scheduled course of treatment, patients could voluntarily continue to complete an additional follow-up survey on WeChat if they continued to receive therapy to monitor the long-term benefits of the real-world application of FEJ. Treatment information was voluntarily completed by the patient and collected by a research assistant at week 8.

The corresponding information was collected at each follow-up visit (Supplementary Table 3). It was used to assess the changes in the TCM symptom evaluation scale of Qi and blood deficiency, the clinical global impression (CGI), the fatigue scale-14 (FS-14), and the Pittsburgh sleep quality index (PSQI) scores before and after treatment with FEJ.

### 2.4. Outcome Measurement

According to the “Guidelines for Clinical Research on New Chinese Medicines” [21], the TCM symptom evaluation scale for Qi and blood deficiency was used to evaluate the improvement of symptoms, including forgetfulness, jaundiced appearance, pallor, slurred speech, shortage of Qi, dizziness, pale mouth and lips, lassitude of spirit, pale nail color, pale eyelids, blurred vision, shortness of breath, lack of strength, spontaneous perspiration, palpitations, menstrual irregularities (any abnormalities in the menstrual cycle, volume, and color), insomnia, limb numbness, and excessive dreaming caused by Qi and blood deficiency. The evaluation of TCM symptoms was divided into 4 grades: cured, improved, no relief, and deteriorated. The CGI scale [22] is a global assessment of the patient's physical health during treatment. The scale has 7 levels: significant deterioration, moderate deterioration, mild deterioration, no remission, mild remission, significant remission, and complete remission. The FS-14 [23] is used to evaluate physical and mental fatigue syndrome. This scale is mainly composed of 14 items divided into two categories: categories 1–8 include 8 items reflecting physical fatigue and categories 9–14 consist of 6 items reflecting mental fatigue. The maximum score is 14 points, and higher scores indicate more severe fatigue. The PSQI [24] was used to assess sleep quality and quantity. The scale contains 19 items across 7 factors. Each factor is scored from 0 to 3, with a total score of 0 to 21. A higher score indicates poorer sleep quality.

### 2.5. Statistical Analysis

Continuous variables are expressed as the mean ± standard deviation (SD) or median with interquartile range (IQR) values. Confidence intervals (CI) are given when necessary. Categorical variables were expressed as frequencies or percentages. Descriptive analyses were performed to summarize patient, disease, and treatment characteristics. For the assessment of changes in the FS-14 and the PSQI from enrollment to the study's predefined follow-up time points, paired *t* tests or Wilcoxon rank sum tests were used. The mean ± SD and *p* values were calculated for paired data. For comparisons between the SF and IDA groups, the independent samples *t* test or Mann–Whitney *U* test was used for quantitative data, and the chi-square test was used for categorical data. Considering the inconsistent baseline levels between the subhealth fatigue (SF) and iron deficiency anemia (IDA) groups, a comparison of FS-14 scores and PSQI scores between the two groups was performed using a general linear model (analysis of covariance) to calibrate the inconsistent baseline variables between the groups (*p* < 0.05). A multivariate logistic regression analysis with CGI scale symptom relief as the dependent variable was developed to analyze the risk factors affecting the degree of relief in QBDS patients. Two-sided statistical tests were used for all analyses. *p* < 0.05 was considered a statistically significant difference. Statistical analyses were performed using SAS (version 9.4).

## 3. Results

### 3.1. Patient Demographics and Baseline Characteristics

**Figure 1:**
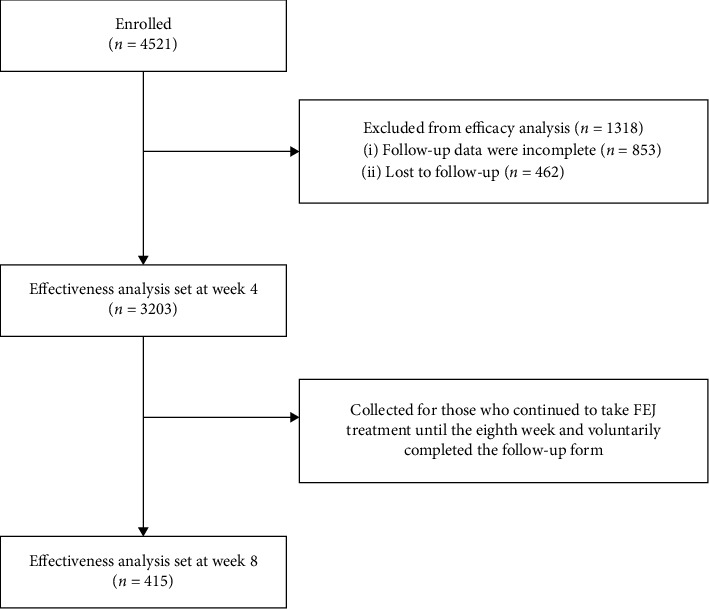
Patient enrollment flowchart.

A total of 4,521 patients were registered during enrollment. The efficacy analysis group included 3,203 patients. A total of 1,318 patients were excluded: 853 individuals did not provide full visit records and 462 were lost to follow-up. All patients (*n* = 3203) who entered the efficacy analysis completed the 4-weekfollow-up. Of these patients, 415 (12.96%) voluntarily completed the 8-weekfollow-up (Figure 1).

The baseline characteristics of patients in the efficacy analysis group are summarized in Table 1. The average age of the participants was 38.34 ± 9.82 years (range 8–93 years); 2,432 (75.92%) were female and 771 (24.08%) were male. The median duration of the disease was 5 days (1, 10). The median number of comorbid TCM symptoms of Qi and blood deficiency was 15 (6, 30). Among the 20 TCM symptoms of Qi and blood deficiency, 12 were present in more than 30% of patients, and the top 3 symptoms were forgetfulness (*n* = 1293; 40.37%), jaundiced appearance (*n* = 1267; 39.56%), and pallor (*n* = 1215; 37.93%) (Table 2). At baseline, the average FS-14 score was 7.62 ± 3.02, and the average PSQI score was 8.35 ± 4.18.

The study included a high proportion of SF and IDA disease diagnoses in QBDS patients, with 2,459 (76.77%) and 713 (22.26%), respectively. The remaining disease diagnoses accounted for less than 3% of the cases, and the small amount of data may lead to unreliable analysis results. Therefore, we stratified the analysis for SF and IDA only. Analysis of the baseline data between the two groups showed that the duration of disease, number of Qi and blood deficiency TCM symptoms, baseline FS-14 score, and baseline PSQI score were significantly lower in the SF group than in the IDA group (*p* < 0.05). There were no significant differences between the two groups in terms of gender and age (*p* > 0.05). These results are shown in Table 3.

### 3.2. Dosing Statistics

The total number of doses taken from baseline to the end of the fourth week of treatment was counted. The results showed that at week four, the total number of doses taken by the patient was 48 (48, 90). At week eight, the total number of doses was 96 (96, 192). The number of doses taken at eight weeks was twice as high as that taken at four weeks.

### 3.3. Effect of FEJ on TCM Symptoms of Qi and Blood Deficiency

After 4 weeks of treatment with FEJ, 3,203 patients were evaluated with the TCM symptom evaluation scale for Qi and blood deficiency; the average remission rate (remission rate is the sum of the cure rate and improvement rate) of the 20 symptoms of Qi and blood deficiency was 92.49%, and 18 symptoms had a remission rate of more than 90%. Among them, the remission rates of lassitude of spirit (95.48%) and dizziness (95.32%) exceeded 95%. At the 8th week, the average remission rate was 94.69%. The remission rate of 19 symptoms exceeded 90%, including 10 symptoms with a remission rate of more than 95%. The remission rates of lassitude of spirit and menstrual irregularities were as high as 99.33% and 98.31%, respectively (Table 4).

### 3.4. Evaluation Results of the CGI Scale after FEJ Treatment

**Figure 2:**
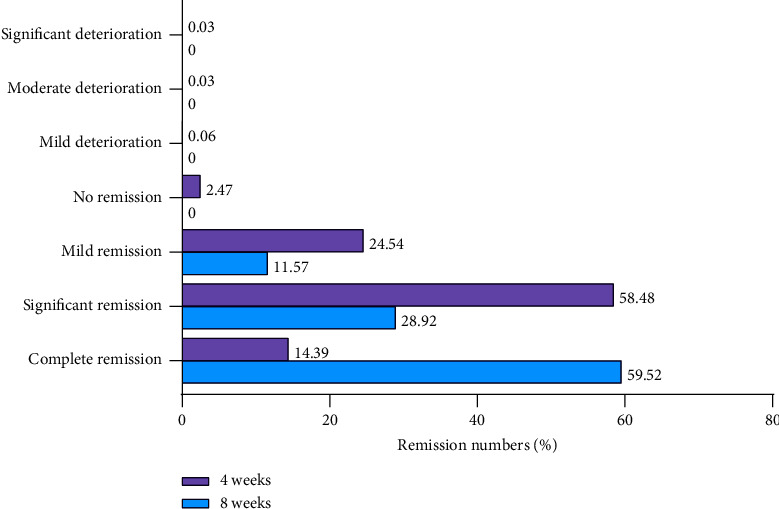
Results of the CGI scale assessment after FEJ treatment.

After 4 weeks of treatment with FEJ, 3,203 patients were evaluated with the CGI scale. The total number of remissions reached 3,120 (97.41%), of which 14.39% of the patients had completely relieved their symptoms and 58.48% had achieved significant subjective relief. A total of 24.54% of patients experienced mild relief of symptoms. A total of 2.47% of patients felt that their symptoms were not relieved and 0.12% of patients experienced deterioration in their symptoms (2 cases with mild deterioration, 1 case with moderate deterioration, and 1 case with significant deterioration). At week 8, all 415 patients felt that their symptoms were in remission, and 59.52% felt that their symptoms had completely resolved. There were no reports of worsening symptoms (Figure 2).

### 3.5. Effect of FEJ Treatment on FS-14 Scores

**Figure 3:**
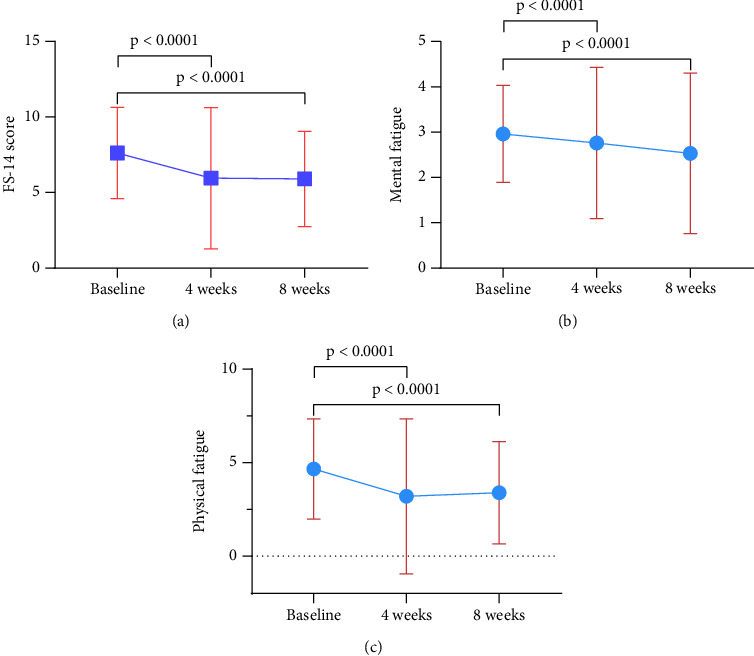
Effect of FEJ treatment on the FS-14 scores. (a) The patients' FS-14 scores after FEJ treatment were significantly lower at weeks 4 and 8 than at baseline. (b) FS-14 mental fatigue scores. (c) FS-14 physical fatigue scores. Statistically significant differences compared to baseline values are represented by *p* values.

After 4 and 8 weeks of treatment with FEJ, there was a statistically significant improvement in the FS-14 total scores and scores on all individual dimensions compared to baseline. The FS-14 total scores decreased by 1.67 ± 4.11 (*p* < 0.001) at week 4 and by 1.72 ± 3.09 (*p* < 0.001) at week 8 (Figure 3(a)). The mental fatigue scores were reduced by 0.2 ± 1.47 (*p* < 0.001) and 0.43 ± 1.54 (*p* < 0.001) at 4 and 8 weeks, respectively, compared to baseline (*p* < 0.001) (Figure 3(b)). The scores of physical fatigue at weeks 4 and 8 decreased by an average of 1.46 ± 3.64 (*p* < 0.001) and 1.27 ± 2.71 (*p* < 0.001), respectively, compared with baseline (Figure 3(c)), with a slight increase in scores at week 8 compared to week 4.

### 3.6. Effect of FEJ Treatment on PSQI Scores

**Figure 4:**
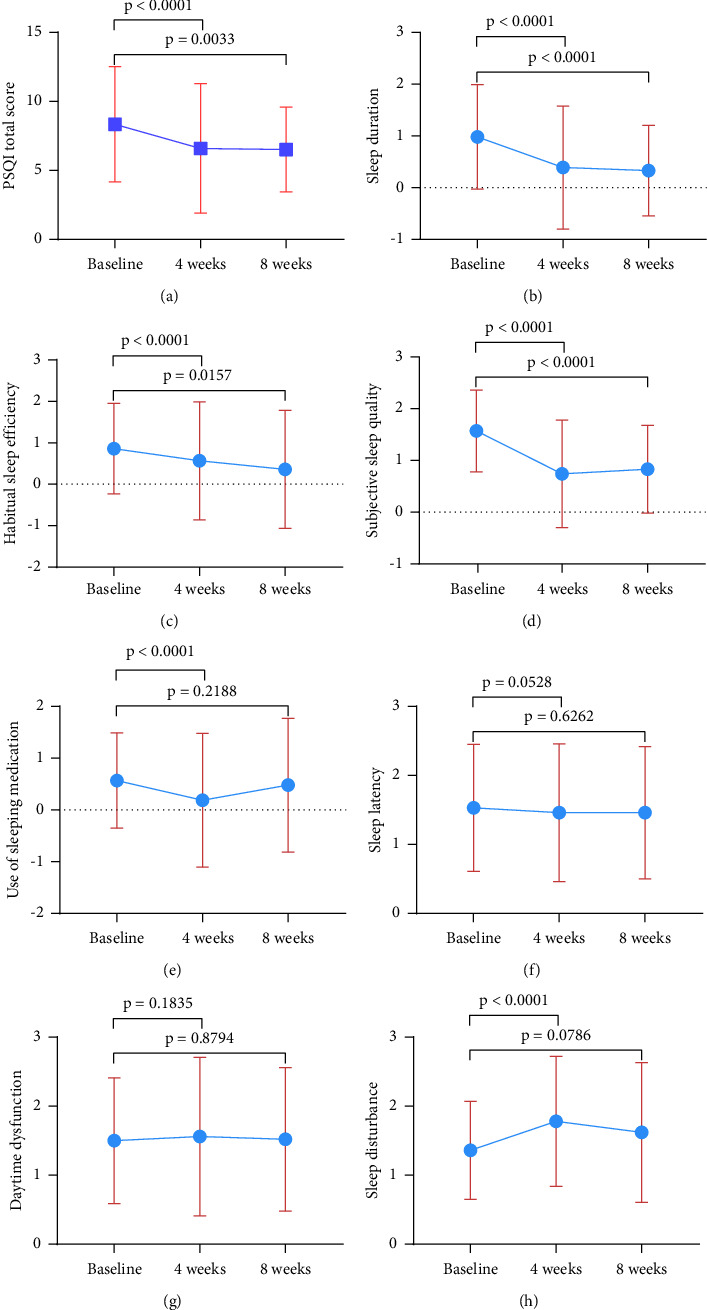
Effect of FEJ treatment on PSQI scores. (a) The patients' PSQI scores after FEJ treatment were significantly lower at weeks 4 and 8 than at baseline. (b) PSQI sleep duration scores. (c) PSQI habitual sleep efficiency scores. (d) PSQI subjective sleep quality scores. (e) PSQI use of sleeping medication scores. (f) PSQI sleep latency scores. (g) PSQI daytime dysfunction scores. (h) PSQI sleep disturbance scores. Statistically significant differences compared to baseline values are represented by *p* values.

The PSQI score of the patients after FEJ treatment was 6.6 ± 4.7 at the 4th week, which was significantly lower than the baseline (*p* < 0.001) (Figure 4(a)). The scores of sleep duration, habitual sleep efficiency, subjective sleep quality, and use of sleeping medication decreased by 0.59 ± 1.11, 0.29 ± 1.29, 0.83 ± 0.94, and 0.38 ± 0.9, respectively, from baseline, which were significantly lower than baseline (*p* < 0.001) (Figures 4(b)–4(e)). The sleep latency improved slightly from baseline (−0.07 ± 0.96, *p* = 0.0528) (Figure 4(f)). The daytime dysfunction score increased slightly but not significantly from baseline (0.06 ± 1.05, *p* = 0.1835) (Figure 4(g)). However, the sleep disturbance score increased significantly from baseline (0.42 ± 0.85, *p* < 0.001) (Figure 4(h)).

A similar change was observed in the data analysis at week 8, with an average score of 6.52 ± 3.07 on the PSQI. Again, the score was significantly lower than that at baseline (*p*=0.0033) (Figure 4(a)). The sleep duration and habitual sleep efficiency scores continued to decrease from week 4, decreasing by 0.65 ± 0.95 (*p* < 0.001) and 0.5 ± 1.29 (*p* < 0.001), respectively, from baseline (Figures 4(b) and 4(c)). The subjective sleep quality score increased slightly from week 4, but the improvement from baseline remained significant (−0.74 ± 0.82, *p* < 0.001) (Figure 4(d)). The score for the use of sleeping medication increased from week 4, although it decreased slightly from baseline (−0.09 ± 0.37, *p*=0.2188) (Figure 4(e)). The sleep latency and daytime dysfunction scores did not change significantly from baseline (−0.07 ± 0.94, *p*=0.6262; 0.02 ± 0.98, *p*=0.8794) (Figures 4(f) and 4(g)), similar to the effects at 4 weeks of treatment. The sleep disturbance score decreased from week 4 but remained slightly higher than the baseline score (0.26 ± 0.9; *p*=0.0786) (Figure 4(h)).

### 3.7. Univariate and Multivariate Analyses of the Outcome of QBDS Patients after FEJ Treatment

Logistic univariate analysis showed that age, disease course, FS-14 score at baseline, and four-week FEJ doses were associated with the degree of symptom relief in patients with QBDS (*p* < 0.05). Furthermore, multivariate logistic analysis was performed on the different indicators in the univariate analysis. The results showed that duration of illness, FS-14 score at baseline, and four-week FEJ doses were independent risk factors for the degree of symptom remission in patients with QBDS (*p* < 0.05) (Tables 5 and 6).

### 3.8. Comparative Analysis of the Efficacy of the SF and IDA Groups

#### 3.8.1. Analysis of the Distribution and Remission of TCM Symptoms of Qi and Blood Deficiency in the SF and IDA Groups

Among the 20 TCM symptoms of Qi and blood deficiency, the top three symptoms in the SF group were forgetfulness (*n* = 864; 38.74%), jaundiced appearance (*n* = 845; 37.89%), and pallor (*n* = 819; 36.73%). After four weeks of FEJ treatment, the average remission rates of the abovementioned three symptoms reached 88.54% (*n* = 765), 90.41% (*n* = 764), and 86.81% (*n* = 711), respectively (Supplementary Table 4). The top three symptoms in the IDA group were insomnia (*n* = 282; 57.67%), pale mouth and lips (*n* = 281; 57.46%), and shortage of Qi (*n* = 280; 57.26%). After four weeks of FEJ treatment, the average remission rates of the three symptoms were 94.33% (*n* = 266), 95.37% (*n* = 268), and 94.29% (*n* = 264), respectively (Supplementary Table 5).

#### 3.8.2. Comparison of Clinical Remission Rates between the SF and IDA Groups

The CGI scale assessment showed that after four weeks of FEJ treatment, total remission reached 2,172 (97.40%) in the SF group and 479 (97.96%) in the IDA group, with no significant difference in the total remission rate between the two groups (*p* > 0.05) (Table 7).

#### 3.8.3. Comparison of FS-14 Scores between the SF and IDA Groups

Patients in the SF and IDA groups had significantly lower FS-14 total scores and scores on all dimensions compared to baseline (*p* < 0.01). An analysis of covariance was applied to calibrate the baseline disease duration, number of TCM symptoms of Qi and blood deficiency, baseline FS-14 scores, and baseline PSQI scores between the SF and IDA groups and then to compare the FS-14 scores between the two groups at four weeks. The results showed that compared to the IDA group, the SF group showed a greater improvement in all dimensions, with the total FS-14 score and physical fatigue dimension score being significantly lower than the IDA group (*p* < 0.01). In contrast, the mental fatigue dimension score difference was not statistically significant (*p* > 0.05) (Table 8).

#### 3.8.4. Comparison of PSQI Scores between the SF and IDA Groups

The total PSQI score and the five dimensions of sleep duration, subjective sleep quality, use of sleeping medication, and sleep disturbance were significantly lower at four weeks of FEJ treatment than at baseline in both the SF and IDA groups (*p* < 0.01).

The habitual sleep efficiency dimension was only improved in the SF group compared to the baseline (*p* < 0.01). The sleep latency dimension was significantly improved only in the IDA group compared to the baseline (*p* < 0.01). There was no significant reduction in daytime dysfunction dimension scores in either group compared to baseline (*p* > 0.05). Variables that were not homogeneous between the two groups were calibrated to compare the difference in PSQI scores between the two groups at four weeks. The results showed that the SF group had a significantly greater reduction in total PSQI scores and habitual sleep efficiency dimension scores than the IDA group (*p* < 0.05). However, no significant differences were found between the two groups in other dimensions at four weeks (*p* > 0.05) (Table 9).

## 4. Discussion

In our study, the CGI scale assessment showed that the number of patients with some symptoms in remission after 4 weeks of treatment with FEJ was 3,120 (97.41%), of which 14.39% were in complete remission. At the 8th week, the number of patients with some symptoms in remission was 415 (100%), of which 59.52% were in complete remission, an increase from the 4th week. At the 4th week of follow-up, the average remission rate of the 20 symptoms of Qi and blood deficiency was 92.49%, which increased to 94.69% at the 8th week. The remission rate gradually increased for two possible reasons: (1) the extended treatment period allowed the FEJ to reach its full efficacy and (2) most patients who adhered to the medication for 8 weeks may have had some improvement at 4 weeks.

The antifatigue effect of FEJ was confirmed in our study. The study showed a continuous decline in total scores on the FS-14 and PSQI scales at four and eight weeks after FEJ treatment. However, the scores on each dimension fluctuated more at eight weeks. Sleep and fatigue are influenced by multiple factors, such as daily activities, life environment, and socioeconomic status [25–28]. Moreover, the patients' follow-up data at the 8th week were reported voluntarily, and we could not know the patients' medication background. Therefore, the data source was not controllable, leading to unreliable results at 8 weeks.

In our study, 76.77% (2,459 cases) of patients were diagnosed with SF. SF is one of the most common types of subhealth, with fatigue as its most prominent manifestation [29]. However, we have not found relevant clinical research evidence on treating SF with FEJ. However, a study on the treatment of women with QBDS with FEJ [30] showed that the brightness of the tongue was reduced, and the color of the tongue and the tongue image improved significantly after treatment with FEJ. It is suggested that FEJ can play a certain therapeutic role in the population with QBDS. This is consistent with the results of our study.

After four weeks of FEJ treatment, patients in the SF and IDA groups showed a significant improvement in symptoms, FS-14 scores, and PSQI scores compared to baseline. The SF group showed a significant reduction in FS-14 and PSQI scores compared to the IDA group. The differences in the baseline disease course, number of symptoms of Qi and blood deficiency, and baseline FS-14 score were more significant between the two groups. In addition, the results of logistic regression analysis in this study showed that disease course, FS-14 score at baseline, and four-week FEJ doses were independent factors affecting the efficacy of FEJ on QBDS. Therefore, we adjusted for inconsistent variables at baseline by analysis of covariance to minimize the effect of confounding factors between the groups.

IDA is the most common form of nutritional anemia and is the ultimate manifestation of iron deficiency in the body, affecting over 1.2 billion people [31]. The symptoms of fatigue in people with IDA are caused by iron deficiency in the brain tissue and are classified as disease fatigue [32]. Therefore, recovery from symptoms is affected by the recovery of ferritin. Recent studies have reported that iron supplementation may take several weeks (>6 weeks) to show complete improvement in fatigue, lethargy, and sleep in IDA patients [33, 34]. Therefore, some experts recommend assessing ferritin response 6–12 weeks after starting oral iron in stable patients without significant anemia [35]. However, SF is not usually abnormal in terms of physiological indicators connected upstream to health and downstream to disease. Therefore, the weaker improvement in fatigue and sleep in the IDA group than in the SF group at four weeks may be related to the shorter follow-up period. It still needs to be determined whether the tonic and blood-generating effects of FEJ will affect the hemoglobin of IDA patients. One study showed [36] that the combination of FEJ significantly improved erythrocytes, hemoglobin, and hematocrit in patients with postpartum anemia compared to iron sucrose alone. Therefore, we recommend the combined use of FEJ and iron supplements in IDA patients to correct iron deficiency and improve symptoms in a shorter time frame.

The observation period of this study was relatively short. However, at the 4th week of follow-up, patients showed a significant improvement in symptoms, indicating that FEJ has a fast onset of action. In the real world, after accounting for factors such as socioeconomics, it is difficult for most patients to adhere to long-term treatment plans. In contrast, FEJ can improve patients' clinical symptoms in a short period, giving FEJ real-world relevance. Second, this study applied the EDC system to record the clinical data of patients. The data collection and recording process are rigorous and scientific, thereby reducing information bias in real-world research. In addition, the results of clinical studies may not always accurately predict real-world outcomes because of limitations such as strict eligibility criteria and unintentional bias produced by the selected study center. Therefore, these observational studies based on patient registration data can well reflect real-world data nationwide and demonstrate the effectiveness of FEJ, thus providing significant research value.

### 4.1. Limitations

There are some limitations in our study. First, this study was observational and did not have a control group, which may have led to selection bias. However, we used continuous recruitment to recruit study participants, thereby minimizing selection bias. Second, the patients voluntarily completed the follow-up information at 8 weeks. Most patients who continued to take the drug for 8 weeks likely achieved some efficacy at 4 weeks. Therefore, the assessment of remission rates at 8 weeks may be biased. In addition, the study results may be influenced by confounding factors, such as diet and concomitant medications.

## 5. Conclusions

Overall, the results of this multicenter, prospective, single-arm, observational study represents the first real-world study of FEJ in China. The study showed that treatment with FEJ effectively improved the clinical symptoms of patients with QBDS.

## Figures and Tables

**Table 1:** Patient demographics and baseline characteristics.

Baselines		Total (*N* = 3203)
Age (years, mean ± SD)		38.34 ± 9.82

*Gender (n, %)*	Male	771 (24.08)
	Female	2432 (75.92)

Disease course at treatment start (days, median, IQR)		5 (1, 10)
Number of TCM symptoms of Qi and blood deficiency (median, IQR)		15 (6, 30)
Baseline FS-14 score (points, mean ± SD)		7.62 ± 3.02
Baseline PSQI score (points, mean ± SD)		8.35 ± 4.18
Current medication (*n*, %)		
Qihekang oral solution		1 (0.03)
*Diagnosis (n, %)*		
SF		2459 (76.77)
IDA		713 (22.26)
Postpartum anemia		90 (2.81)
Leukopenia		30 (0.94)
Anemia caused by uterine bleeding		10 (0.31)
Cancer-related fatigue		8 (0.25)
Chemotherapy-associated anemia		3 (0.09)
Post chemotherapy myelosuppression		2 (0.06)
*Medical history (n, %)*		
Anemia		7 (0.21)
Hypertension		4 (0.12)
Diabetes mellitus		2 (0.06)
Lung cancer		2 (0.06)
Disc disease		2 (0.06)
Mouth ulcers		2 (0.06)
Rhinitis		1 (0.03)
Liver cirrhosis		1 (0.03)
Hypoglycemia		1 (0.03)
Chronic gastritis		1 (0.03)
Ligament injury		1 (0.03)
Appendectomy		1 (0.03)
Cerebral infarction		1 (0.03)
Interstitial pulmonary fibrosis		1 (0.03)
Rheumatoid arthritis		1 (0.03)
*Medication history (n, %)*		
Iron supplements		1 (0.03)
Nifedipine extended-release tablets		1 (0.03)
FEJ		7 (0.21)
Liuwei Dihuang pills		1 (0.03)
Other patented Chinese medicine prescriptions that replenish Qi and nourish blood		6 (0.19)

SD, standard deviation; IQR, interquartile range; TCM, traditional Chinese medicine; FS-14, fatigue scale-14; PSQI, Pittsburgh sleep quality index; SF, subhealth fatigue; IDA, iron deficiency anemia; and FEJ, Fufang E'jiao Jiang. Values are presented as the mean ± SD, *n* (%), and median (IQR) values. Other patented Chinese medicine prescriptions for replenishing Qi and nourishing blood include Anshen Yangxue oral liquid, Yang Xueyin, Yiqi Buxue oral liquid, and Shengxuebao mixture.

**Table 2:** Presence of symptoms of Qi and blood deficiency at baseline.

TCM symptoms	*N* (%)
Forgetfulness	1293 (40.37)
Jaundiced appearance	1267 (39.56)
Pallor	1215 (37.93)
Slurred speech	1195 (37.31)
Shortage of Qi	1194 (37.28)
Dizziness	1154 (36.03)
Pale mouth and lips	1140 (35.59)
Lassitude of spirit	1129 (35.25)
Pale nail color	1035 (32.31)
Pale eyelids	1025 (32.00)
Blurred vision and dizziness	1020 (31.85)
Shortness of breath	992 (30.97)
Lack of strength	948 (29.60)
Spontaneous perspiration	942 (29.41)
Blurred vision	916 (28.60)
Palpitation	886 (27.66)
Menstrual irregularities	792 (24.73)
Insomnia	757 (23.63)
Limb numbness	749 (23.38)
Excessive dreaming	746 (23.29)

TCM, traditional Chinese medicine. Data are expressed as *N* (%).

**Table 3:** Comparison of baseline data between the SF and IDA groups.

Information		SF group (*n* = 2230)	IDA group (*n* = 489)	*p*
*Gender (n, %)*	Male	541 (24.26)	125 (25.56)	0.544
	Female	1689 (75.74)	364 (74.44)	

Age (years, mean ± SD)		39.58 ± 11.22	39.86 ± 10.60	0.321
Disease course at treatment start (days, median, IQR)		5.5 (7, 30)	15 (5, 20)	<0.001
Number of symptoms of Qi and blood deficiency (median, IQR)		4 (1, 9)	9 (2, 15)	<0.001
Baseline FS-14 score (points, mean ± SD)		7.60 ± 3.00	8.40 ± 2.98	<0.001
Baseline PSQI score (points, mean ± SD)		8.03 ± 4.12	9.49 ± 3.90	<0.001

SF, subhealth fatigue; IDA, iron deficiency anemia; SD, standard deviation; IQR, interquartile range; FS-14, fatigue scale-14; PSQI, Pittsburgh sleep quality index. Values are presented as the mean ± SD, *n* (%), or median (IQR) values.

**Table 4:** Results of the TCM symptom evaluation scale of Qi and blood deficiency after FEJ treatment.

	*4 weeks (N* *=* *3203)*					*8 weeks (N* *=* *415)*				
	Cured (*n,* %)	Improved (*n,* %)	No relief (*n,* %)	Deterioration (*n,* %)	Remission rate (*n,* %)	Cured (*n,* %)	Improved (*n,* %)	No relief (*n,* %)	Deterioration (*n,* %)	Remission rate (*n,* %)
Lassitude of spirit	322 (28.52)	756 (66.96)	49 (4.34)	2 (0.18)	1078 (95.48)	95 (63.76)	53 (35.57)	1 (0.67)	0 (0.00)	148 (99.33)
Lack of strength	250 (26.37)	635 (66.98)	63 (6.65)	0 (0.00)	885 (93.35)	78 (62.90)	43 (34.68)	3 (2.42)	0 (0.00)	121 (97.58)
Shortage of Qi	360 (30.15)	768 (64.32)	65 (5.44)	1 (0.08)	1128 (94.47)	107 (73.29)	34 (23.29)	5 (3.42)	0 (0.00)	141 (96.58)
Laziness speech	379 (31.72)	741 (62.01)	75 (6.28)	0 (0.00)	1120 (93.73)	102 (75.00)	28 (20.59)	6 (4.41)	0 (0.00)	130 (95.59)
Shortness of breath	313 (31.55)	614 (61.90)	65 (6.55)	0 (0.00)	927 (93.45)	83 (77.57)	21 (19.63)	3 (2.80)	0 (0.00)	104 (97.20)
Dizziness	341 (29.55)	759 (65.77)	54 (4.68)	0 (0.00)	1100 (95.32)	96 (76.19)	27 (21.43)	3 (2.38)	0 (0.00)	123 (97.62)
Dizzy vision	305 (29.90)	641 (62.84)	73 (7.16)	1 (0.10)	946 (92.74)	82 (75.23)	23 (21.10)	4 (3.67)	0 (0.00)	105 (96.33)
Spontaneous perspiration	272 (28.87)	593 (62.95)	77 (8.17)	0 (0.00)	865 (91.82)	65 (70.65)	18 (19.57)	9 (9.78)	0 (0.00)	83 (90.22)
Pale face	328 (27.00)	761 (62.63)	126 (10.37)	0 (0.00)	1089 (89.63)	102 (66.67)	29 (18.95)	22 (14.38)	0 (0.00)	131 (85.62)
Yellowish face	342 (26.99)	820 (64.72)	105 (8.29)	0 (0.00)	1162 (91.71)	103 (66.88)	40 (25.97)	11 (7.14)	0 (0.00)	143 (92.85)
Pale eyelid color	290 (28.29)	648 (63.22)	87 (8.49)	0 (0.00)	938 (91.51)	74 (63.79)	32 (27.59)	10 (8.62)	0 (0.00)	106 (91.38)
Pale mouth and lips	319 (27.98)	741 (65.00)	80 (7.02)	0 (0.00)	1060 (92.98)	83 (63.85)	40 (30.77)	7 (5.38)	0 (0.00)	123 (94.62)
Light nail color	283 (27.34)	680 (65.70)	71 (6.86)	1 (0.10)	963 (93.04)	74 (62.18)	40 (33.61)	5 (4.20)	0 (0.00)	114 (95.79)
Blurred vision	243 (26.53)	595 (64.96)	78 (8.52)	0 (0.00)	838 (91.49)	63 (68.48)	24 (26.09)	5 (5.43)	0 (0.00)	87 (94.57)
Palpitation	236 (26.64)	580 (65.46)	70 (7.90)	0 (0.00)	816 (92.10)	61 (60.40)	34 (33.66)	6 (5.94)	0 (0.00)	95 (94.06)
Insomnia	218 (28.80)	468 (61.82)	71 (9.38)	0 (0.00)	686 (90.62)	64 (64.00)	31 (31.00)	5 (5.00)	0 (0.00)	95 (95.00)
Limb numbness	173 (23.10)	514 (68.62)	62 (8.28)	0 (0.00)	687 (91.72)	49 (63.64)	23 (29.87)	5 (6.49)	0 (0.00)	72 (93.51)
Excessive dreaming	196 (26.27)	483 (64.75)	67 (8.98)	0 (0.00)	679 (91.02)	58 (62.37)	29 (31.18)	6 (6.45)	0 (0.00)	87 (93.55)
Forgetfulness	312 (24.13)	851 (65.82)	130 (10.05)	0 (0.00)	1163 (89.95)	93 (62.00)	48 (32.00)	9 (6.00)	0 (0.00)	141 (94.00)
Menstrual irregularities	247 (31.23)	494 (62.45)	50 (6.32)	0 (0.00)	741 (93.68)	67 (56.78)	49 (41.53)	2 (1.69)	0 (0.00)	116 (98.31)

TCM, traditional Chinese medicine; FEJ, Fufang E'jiao Jiang. Data are expressed as *n* (%).

**Table 5:** Argument assignment table.

Factors	Assignment values
Age (years)	≤65 = 0; >65 = 1
Disease course at treatment start (days)	<30 = 0; ≥30 = 1
Number of symptoms of Qi and blood deficiency	≤10 = 0; >10 = 1
Baseline FS-14 score	Continuous
Baseline PSQI score	Continuous
Total amount of FEJ taken at four weeks	Continuous

FS-14, fatigue scale-14 and PSQI, Pittsburgh sleep quality index.

**Table 6:** Univariate and multivariate logistic regression analyses of the degree of remission in QBDS patients treated with FEJ.

Indicators	*Single-factor*			*Multifactor*		
	OR	95% CI	*p*	OR	95% CI	*p*
Age	7.542	3.340∼17.031	0.001	—	—	—
Male	0.890	0.650∼1.218	0.466	—	—	—
Number of symptoms of Qi and blood deficiency	1.090	0.809∼1.468	0.570	—	—	—
Disease course at treatment start	1.412	1.055∼1.888	0.020	1.379	1.025∼1.854	0.034
Baseline FS-14 score	1.088	1.031∼1.149	0.002	1.070	1.012∼1.130	0.016
Baseline PSQI score	1.034	0.999∼1.071	0.058	—	—	—
Total amount of FEJ taken at four weeks	0.987	0.982∼0.992	0.001	0.987	0.982∼0.993	0.001

QBDS, Qi and blood deficiency syndrome; FEJ, Fufang E'jiao Jiang; OR, odds ratio; CI, confidence interval; FS-14, fatigue scale-14; and PSQI, Pittsburgh sleep quality index.

**Table 7:** Comparison of clinical remission rates between the SF and IDA groups.

Groups	*N*	Complete remission (*n,* %)	Significant remission (*n,* %)	Mild remission (n, %)	No remission (*n,* %)	Mild deterioration (*n,* %)	Moderate deterioration (*n,* %)	Significant deterioration (*n,* %)	Total remission(*n,* %)
SF group	2230	338 (15.16)	1274 (57.13)	560 (25.11)	56 (2.51)	0 (0.00)	1 (0.04)	1 (0.04)	2172 (97.40)
IDA group	489	62 (12.68)	304 (62.17)	113 (23.11)	9 (1.84)	1 (0.20)	0 (0.00)	0 (0.00)	479 (97.96)
*F*									2.826
*p*									0.243

SF, subhealth fatigue and IDA, iron deficiency anemia. Data are expressed as *n* (%).

**Table 8:** Comparison of the FS-14 scores after four weeks of FEJ treatment in the SF and IDA groups after calibration for covariance.

	*Baseline*	*4 weeks*	*Adjust the four-week score after the baseline*			*F value*	*p value*
	Mean ± SD	Mean ± SD	Mean	SE	95 CI		
*FS-14 scores*						14.846	<0.001
SF group (2230)	7.60 ± 3.00	5.74 ± 3.49^▲^	5.83	0.13	[5.58, 6.08]		
IDA group (489)	8.40 ± 2.98^*∗∗*^	7.01 ± 3.65^▲^^*∗∗*^	6.77^*∗∗*^	0.21	[6.37, 7.17]		
*Mental fatigue*						0.035	0.853
SF group (2230)	2.99 ± 1.16	2.78 ± 1.14^▲^	2.78	0.04	[2.70, 2.86]		
IDA group (489)	2.98 ± 1.06	2.77 ± 0.96^▲^	2.76	0.06	[2.64, 2.89]		
*Physical fatigue*						18.359	<0.001
SF group (2230)	4.61 ± 2.57	2.96 ± 3.15^▲^	3.06	0.12	[2.83, 3.28]		
IDA group (489)	5.42 ± 2.69^*∗∗*^	4.24 ± 3.46^▲^^*∗∗*^	4.00^*∗∗*^	0.19	[3.64, 4.37]		

FS-14, fatigue scale-14; FEJ, Fufang E'jiao Jiang; SF, subhealth fatigue; IDA, iron deficiency anemia; SD, standard deviation; SE, standard error; and CI, confidence interval. Data are expressed as the mean, mean ± SD, SE, or 95% CI. Compared with the same group at baseline, ^△^*p* < 0.05, ^▲^*p* < 0.01; with the same time point in the SF group, ^*∗*^*p* < 0.05, ^*∗∗*^*p* < 0.01.

**Table 9:** Comparison of PSQI scores after four weeks of FEJ treatment in the SF and IDA groups after calibration for covariance.

	*Baseline*	*4 weeks*	*Adjust the four-week score after the baseline*			*F values*	*p values*
	Mean ± SD	Mean ± SD	Mean	SE	95 CI		
*PSQI scores*						4.493	0.034
SF group (2230)	8.03 ± 4.12	6.60 ± 3.20^▲^	6.70	0.13	[6.46, 6.97]		
IDA group (489)	9.49 ± 3.90^*∗∗*^	7.54 ± 3.21^▲^^*∗∗*^	7.26^*∗*^	0.22	[6.83, 7.68]		
*Sleep duration*						1.847	0.175
SF group (2230)	0.92 ± 1.00	0.36 ± 0.71^▲^	0.37	0.03	[0.31, 0.43]		
IDA group (489)	1.11 ± 0.95^*∗*^	0.48 ± 0.73^▲^^*∗*^	0.45	0.05	[0.35, 0.54]		
*Habitual sleep efficiency*						6.272	0.012
SF group (2230)	0.79 ± 1.05	0.62 ± 0.95^▲^	0.64	0.04	[0.55, 0.72]		
IDA group (489)	1.01 ± 1.16^*∗*^	0.89 ± 1.05^*∗∗*^	0.84^*∗*^	0.07	[0.70, 0.97]		
*Subjective sleep quality*						1.289	0.257
SF group (2230)	1.53 ± 0.81	0.71 ± 0.70^▲^	0.72	0.03	[0.67, 0.78]		
IDA group (489)	1.73 ± 0.66^*∗∗*^	0.81 ± 0.58^▲^	0.78	0.05	[0.69, 0.87]		
*Use of sleeping medication*						1.401	0.237
SF group (2230)	0.48 ± 0.90	0.14 ± 0.48^▲^	0.15	0.02	[0.12, 0.19]		
IDA group (489)	0.71 ± 0.91^*∗∗*^	0.25 ± 0.48^▲^^*∗∗*^	0.20	0.03	[0.14, 0.26]		
*Sleep latency*						3.152	0.076
SF group (2230)	1.49 ± 0.92	1.44 ± 0.60	1.46	0.03	[1.41, 1.50]		
IDA group (489)	1.77 ± 0.94^*∗∗*^	1.58 ± 0.62^▲^^*∗∗*^	1.54	0.04	[1.46, 1.62]		
*Daytime dysfunction*						1.619	0.204
SF group (2230)	1.52 ± 0.95	1.57 ± 0.68	1.59	0.03	[1.53, 1.64]		
IDA group (489)	1.66 ± 0.82	1.69 ± 0.63^*∗*^	1.66	0.05	[1.57, 1.75]		
*Sleep disturbance*						0.188	0.665
SF group (2230)	1.31 ± 0.71	1.77 ± 0.60^▲^	1.79	0.03	[1.74, 1.83]		
IDA group (489)	1.51 ± 0.61^*∗∗*^	1.84 ± 0.56^▲^	1.81	0.04	[1.73, 1.89]		

PSQI, Pittsburgh sleep quality index; FEJ, Fufang E'jiao Jiang; SF, subhealth fatigue; IDA, iron deficiency anemia; SD, standard deviation; SE, standard error; and CI, confidence interval. Data are expressed as the mean, mean ± SD, SE, or 95% CI. Compared with the same group at baseline, ^△^*p* < 0.05, ^▲^*p* < 0.01; with the same time point in the SF group, ^*∗*^*p* < 0.05, ^*∗∗*^*p* < 0.01.

## Data Availability

The data used to support the findings of this study are available from the corresponding author upon request.

## Abbreviations

FEJ: Fufang E'jiao Jiang
QBDS: Qi and blood deficiency syndrome
TCM: Traditional Chinese medicine
CGI: Clinical global impression
FS-14: Fatigue scale-14
PSQI: Pittsburgh sleep quality index
CFDA: China Food and Drug Administration
ChiCTR: Chinese Clinical Trials Registry
STROBE: Strengthening the Reporting of Observational Studies in Epidemiology
ECD: Electronic data capture
CRF: Case report form
IQR: Interquartile range
SD: Standard deviations
SF: Subhealth fatigue
IDA: Iron deficiency anemia.

## Supplementary Materials

Supplementary Materials Supplementary Table 1. STROBE Statement—checklist. Supplementary Table 2. TCM diagnostic criteria for QBDS. Supplementary Table 3. Follow-up plan. Supplementary Table 4. Distribution and remission of TCM symptoms of Qi and blood deficiency in the SF group at four weeks. Supplementary Table 5. Distribution and remission of TCM symptoms of Qi and blood deficiency in the IDA group at four weeks.
