# Supplementary material for: Efficacy of Fufang E'jiao Jiang in the Treatment of Patients with Qi and Blood Deficiency Syndrome: A Real-World Prospective Multicenter Study with a Patient Registry
Source: Evid Based Complement Alternat Med. 2023 Feb 3;2023:3179489. doi: 10.1155/2023/3179489 (PMC9918352; doi:10.1155/2023/3179489)
Supplement: Supplementary Materials — Supplementary Table 1. STROBE Statement—checklist. Supplementary Table 2. TCM diagnostic criteria for QBDS. Supplementary Table 3. Follow-up plan. Supplementary Table 4. Distribution and remission of TCM symptoms of Qi and blood deficiency in the SF group at four weeks. Supplementary Table 5. Distribution and remission of TCM symptoms of Qi and blood deficiency in the IDA group at four weeks. [file 3179489.f1.zip › Supplementary Table 2.docx]

Supplementary Table 2: TCM diagnostic criteria for QBDS.

| Primary symptom | Lassitude of Spirit and Lack of Strength. |
| --- | --- |
| Secondary symptoms | Pale White or Yellowish Face, Shortage of Qi, Slurred Speech, Dizziness, Spontaneous Perspiration, Palpitation. |
| Tongue Manifestation | Pale Tongue or Tooth-Marked Tongue. |
| Pulse Condition | Fine Pulse, Weak Pulse or Vacuous Pulse. |
| The diagnosis of QBDS must include both the primary symptom and one additional symptom from the three categories of secondary symptoms, Tongue Manifestation and Pulse Condition. | |

TCM, traditional Chinese medicine; QBDS, Qi and blood deficiency syndrome.
