# Supplementary material for: Efficacy of Fufang E'jiao Jiang in the Treatment of Patients with Qi and Blood Deficiency Syndrome: A Real-World Prospective Multicenter Study with a Patient Registry
Source: Evid Based Complement Alternat Med. 2023 Feb 3;2023:3179489. doi: 10.1155/2023/3179489 (PMC9918352; doi:10.1155/2023/3179489)
Supplement: Supplementary Materials — Supplementary Table 1. STROBE Statement—checklist. Supplementary Table 2. TCM diagnostic criteria for QBDS. Supplementary Table 3. Follow-up plan. Supplementary Table 4. Distribution and remission of TCM symptoms of Qi and blood deficiency in the SF group at four weeks. Supplementary Table 5. Distribution and remission of TCM symptoms of Qi and blood deficiency in the IDA group at four weeks. [file 3179489.f1.zip › Supplementary Table 3.docx]

Supplementary Table 3: Follow-up plan.

| Project | Baseline | Follow-up 1 | Follow-up 2 |
| --- | --- | --- | --- |
|  | Day 0 | For 4 weeks | For 8 weeks |
| Informed consent | × |  |  |
| Patient demographic | × |  |  |
| Disease information | × |  |  |
| Vital signs | × | × | × |
| Number of drugs purchased | × | × | × |
| TCM Symptom Evaluation Scale for Qi and Blood Deficiency | × | × | × |
| CGI scale |  | × | × |
| FS-14 | × | × | × |
| PSQI | × | × | × |
| Concomitant therapy | × | × | × |

TCM, traditional Chinese medicine; CGI, Clinical Global Impression; FS-14, Fatigue Scale-14; PSQI, Pittsburgh Sleep Quality Index.

Four weeks was the designated preset follow-up time; those who took medication for more than 4 weeks could voluntarily continue to fill out the follow-up information collection form on the public website on WeChat. Treatment information voluntarily completed by the patient was collected by the research assistant at week 8.
