# Supplementary material for: Efficacy of Fufang E'jiao Jiang in the Treatment of Patients with Qi and Blood Deficiency Syndrome: A Real-World Prospective Multicenter Study with a Patient Registry
Source: Evid Based Complement Alternat Med. 2023 Feb 3;2023:3179489. doi: 10.1155/2023/3179489 (PMC9918352; doi:10.1155/2023/3179489)
Supplement: Supplementary Materials — Supplementary Table 1. STROBE Statement—checklist. Supplementary Table 2. TCM diagnostic criteria for QBDS. Supplementary Table 3. Follow-up plan. Supplementary Table 4. Distribution and remission of TCM symptoms of Qi and blood deficiency in the SF group at four weeks. Supplementary Table 5. Distribution and remission of TCM symptoms of Qi and blood deficiency in the IDA group at four weeks. [file 3179489.f1.zip › Supplementary Table 4.docx]

Supplementary Table 4: Distribution and remission of TCM symptoms of Qi and blood deficiency in the SF group at four weeks.

| TCM symptoms | SF Group (*n,* %) | Cured  (*n,* %) | Improved  (*n,* %) | No relief  (*n,* %) | Deterioration  (*n,* %) | Remission rate  (*n,* %) |
| --- | --- | --- | --- | --- | --- | --- |
| Forgetfulness | 864 (38.74) | 234 (27.08) | 531 (61.46) | 99 (11.46) | 0 (0.00) | 765 (88.54) |
| Jaundiced Appearance | 845 (37.89) | 251 (29.70) | 513 (60.71) | 81 (9.59) | 0 (0.00) | 764 (90.41) |
| Pallor | 819 (36.73) | 234 (28.57) | 477 (58.24) | 108 (13.19) | 0 (0.00) | 711 (86.81) |
| Slurred Speech | 799 (35.83) | 282 (35.29) | 466 (58.32) | 51 (6.38) | 0 (0.00) | 748 (93.62) |
| Shortage of Qi | 781 (35.02) | 262 (33.55) | 479 (61.33) | 40 (5.12) | 0 (0.00) | 741 (94.88) |
| Dizziness | 748 (33.54) | 226 (30.21) | 418 (55.88) | 104 (13.90) | 0 (0.00) | 644 (86.10) |
| Lassitude of Spirit | 746 (33.45) | 227 (30.43) | 485 (65.01) | 33 (4.42) | 1 (0.13) | 712 (95.44) |
| Pale Mouth and Lips | 734 (32.91) | 232 (31.61) | 445 (60.63) | 57 (7.77) | 0 (0.00) | 677 (92.23) |
| Pale Nail Color | 659 (29.55) | 207 (31.41) | 404 (61.31) | 47 (7.13) | 1 (0.15) | 611 (92.72) |
| Pale Eyelids | 652 (29.24) | 211 (32.36) | 375 (57.52) | 66 (10.12) | 0 (0.00) | 586 (89.88) |
| Blurred Vision and Dizziness | 642 (28.79) | 214 (33.33) | 377 (58.72) | 51 (7.94) | 0 (0.00) | 591 (92.06) |
| Shortness of Breath | 632 (28.34) | 228 (36.08) | 357 (56.49) | 47 (7.44) | 0 (0.00) | 585 (92.56) |
| Lack of Strength | 629 (28.21) | 183 (29.09) | 397 (63.12) | 49 (7.79) | 0 (0.00) | 580 (92.21) |
| Spontaneous Perspiration | 591 (26.50) | 190 (32.15) | 342 (57.87) | 59 (9.98) | 0 (0.00) | 532 (90.02) |
| Blurred Vision | 571 (25.61) | 182 (31.87) | 341 (59.72) | 48 (8.41) | 0 (0.00) | 523 (91.59) |
| Palpitation | 543 (24.35) | 175 (32.23) | 322 (59.30) | 46 (8.47) | 0 (0.00) | 497 (91.53) |
| Insomnia | 503 (22.56) | 170 (33.80) | 284 (56.46) | 49 (9.74) | 0 (0.00) | 454 (90.26) |
| Menstrual Irregularities | 492 (22.06) | 155 (31.50) | 302 (61.38) | 35 (7.11) | 0 (0.00) | 457 (92.89) |
| Excessive Dreaming | 491 (22.02) | 143 (29.12) | 297 (60.49) | 51 (10.39) | 0 (0.00) | 440 (89.61) |
| Limb Numbness | 455 (20.40) | 121 (26.59) | 290 (63.74) | 44 (9.67) | 0 (0.00) | 411 (90.33) |

TCM, traditional Chinese medicine; SF, subhealth fatigue. Data are expressed as *n* (%).
