# Supplementary material for: Efficacy of Fufang E'jiao Jiang in the Treatment of Patients with Qi and Blood Deficiency Syndrome: A Real-World Prospective Multicenter Study with a Patient Registry
Source: Evid Based Complement Alternat Med. 2023 Feb 3;2023:3179489. doi: 10.1155/2023/3179489 (PMC9918352; doi:10.1155/2023/3179489)
Supplement: Supplementary Materials — Supplementary Table 1. STROBE Statement—checklist. Supplementary Table 2. TCM diagnostic criteria for QBDS. Supplementary Table 3. Follow-up plan. Supplementary Table 4. Distribution and remission of TCM symptoms of Qi and blood deficiency in the SF group at four weeks. Supplementary Table 5. Distribution and remission of TCM symptoms of Qi and blood deficiency in the IDA group at four weeks. [file 3179489.f1.zip › Supplementary Table 5.docx]

Supplementary Table 5: Distribution and remission of TCM symptoms of Qi and blood deficiency in the IDA group at four weeks.

| TCM symptoms | IDA Group (*n,* %) | Cured  (*n,* %) | Improved  (*n,* %) | No relief  (*n,* %) | Deterioration  (*n,* %) | Remission rate  (*n,* %) |
| --- | --- | --- | --- | --- | --- | --- |
| Insomnia | 282 (57.67) | 45 (15.96) | 221 (78.37) | 16 (5.67) | 0 (0.00) | 266 (94.33) |
| Pale Mouth and Lips | 281 (57.46) | 59 (21.00) | 209 (74.38) | 13 (4.63) | 0 (0.00) | 268 (95.37) |
| Shortage of Qi | 280 (57.26) | 68 (24.29) | 196 (70.00) | 15 (5.36) | 1 (0.36) | 264 (94.29) |
| Dizziness | 267 (54.60) | 62 (23.22) | 196 (73.41) | 9 (3.37) | 0 (0.00) | 258 (96.63) |
| Jaundiced appearance | 267 (54.60) | 56 (20.97) | 198 (74.16) | 13 (4.87) | 0 (0.00) | 254 (95.13) |
| Pallor | 262 (53.58) | 62 (23.66) | 189 (72.14) | 11 (4.20) | 0 (0.00) | 251 (95.80) |
| Blurred Vision and Dizziness | 262 (53.58) | 63 (24.05) | 185 (70.61) | 13 (4.96) | 1 (0.38) | 248 (94.66) |
| Pale Nail Color | 261 (53.37) | 48 (18.39) | 198 (75.86) | 15 (5.75) | 0 (0.00) | 246 (94.25) |
| Slurred Speech | 254 (51.94) | 63 (24.80) | 179 (70.47) | 12 (4.72) | 0 (0.00) | 242 (95.28) |
| Shortness of Breath | 252 (51.53) | 59 (23.41) | 182 (72.22) | 11 (4.37) | 0 (0.00) | 241 (95.63) |
| Spontaneous perspiration | 249 (50.92) | 54 (21.69) | 183 (73.49) | 12 (4.82) | 0 (0.00) | 237 (95.18) |
| Pale Eyelids | 247 (50.51) | 51 (20.65) | 187 (75.71) | 9 (3.64) | 0 (0.00) | 238 (96.36) |
| Blurred Vision | 237 (48.47) | 42 (17.72) | 176 (74.26) | 19 (8.02) | 0 (0.00) | 218 (91.98) |
| Palpitation | 235 (48.06) | 37 (15.74) | 186 (79.15) | 12 (5.11) | 0 (0.00) | 223 (94.89) |
| Lassitude of Spirit | 212 (43.35) | 59 (27.83) | 140 (66.04) | 12 (5.66) | 1 (0.47) | 199 (93.87) |
| Limb Numbness | 200 (40.90) | 34 (17.00) | 156 (78.00) | 10 (5.00) | 0 (0.00) | 190 (95.00) |
| Menstrual Irregularities | 183 (37.42) | 1 (0.55) | 52 (28.42) | 122 (66.67) | 8 (4.37) | 53 (28.96) |
| Lack of Strength | 163 (33.33) | 35 (21.47) | 120 (73.62) | 8 (4.91) | 0 (0.00) | 155 (95.09) |
| Excessive Dreaming | 148 (30.27) | 29 (19.59) | 112 (75.68) | 7 (4.73) | 0 (0.00) | 141 (95.27) |
| Insomnia | 141 (28.83) | 24 (17.02) | 109 (77.30) | 8 (5.67) | 0 (0.00) | 133 (94.33) |

TCM, traditional Chinese medicine; IDA, iron deficiency anemia. Data are expressed as *n* (%).
